# Molecular epidemiological characteristics of carbapenem-resistant *Enterobacter cloacae complex*

**DOI:** 10.1038/s41598-026-55757-6

**Published:** 2026-05-29

**Authors:** Jing He, Kuo Cheng, Qian Sui, Junhua Feng, Xiaoyu Zhang, Kaixuan Zhao, Wei Wang, Yanli Deng

**Affiliations:** 1 Department of Clinical Laboratory, Fourth Hospital of Hebei Medical University, Shijiazhuang, China; 2 Department of Clinical Laboratory, Xingtai People’s Hospital, Xingtai, China

**Keywords:** *Enterobacter cloacae complex*, Carbapenemase, Plasmid, Virulence gene, Epidemiology, Clinical microbiology, Policy and public health in microbiology, Antimicrobial resistance

## Abstract

This study aimed to monitor the evolving antibiotic resistance patterns of carbapenem-resistant *Enterobacter cloacae complex* (CR-ECC) at the Fourth Hospital of Hebei Medical University, characterize the molecular epidemiology through resistance gene profiling and phylogenetic analysis, investigate virulence gene profiles, and establish an evidence-based framework for optimizing clinical antimicrobial therapy and infection control strategies. 32 CR-ECC isolates (2018–2022) underwent Vitek-based identification and susceptibility testing. Carbapenemase production was confirmed by mCIM, with whole-genome sequencing (Illumina) for MLST phylogeny (MEGA/iTOL). Plasmid replicons and resistance/virulence genes were analyzed using PlasmidFinder and RGI/BLAST respectively. CR-ECC emerged in 2020, reaching 32 cases by 2022 as the third most prevalent carbapenem-resistant *Enterobacteriaceae*. Isolates mainly from sputum (43.75%, 14/32) and blood (25.00%, 8/32), with 53.13% ICU origins. Universal resistance to piperacillin/tazobactam, ceftazidime, imipenem; full susceptibility to tigecycline/colistin. Molecular analysis identified 8 STs, predominantly ST171 (56.25%) carrying *blaNDM-1/OXA-1*. Plasmid analysis revealed Col(pHAD28)/IncC dominance with ST-specific distribution. Virulence genes showed near-universal *fimA/csgD/entB* (96.88%) and *mrkD* (90.63%). These findings highlight the therapeutic potential of tigecycline, colistin, and amikacin against CR-ECC, while emphasizing strain-specific aztreonam susceptibility in metallo-β-lactamase producers.

## Introduction

*Enterobacter cloacae complex* (ECC), comprising clinically significant species such as *Enterobacter cloacae*, *E. hormaechei*, and *E. asburiae*, represents a major opportunistic pathogen group within *Enterobacteriaceae* in China, consistently ranking among the top three most frequently isolated clinical *Enterobacteriaceae*. These organisms demonstrate broad pathogenic potential, causing multi-system infections ranging from respiratory to urogenital diseases, making accurate identification critical in anti-infective management. Of particular concern is their escalating antibiotic resistance, exemplified by an alarming 3.5-fold surge in imipenem resistance rates from 2.8% (2011) to 9.8% (2023) according to the China Antimicrobial Surveillance Network. Our five-year molecular epidemiological investigation employs advanced detection methodologies - including whole-genome sequencing and resistance gene profiling - to systematically characterize ECC’s evolutionary patterns. This comprehensive approach not only expands the molecular database for ECC but also strengthens antimicrobial resistance surveillance through detailed molecular epidemiology, ultimately providing critical insights to optimize empirical therapy guidelines and inform precision treatment strategies against this increasingly drug-resistant pathogen group.

## Materials and methods

### Source of strains

This five-year retrospective study (2018–2022) analyzed 4,101 *Enterobacteriaceae* collected from clinical departments at the Fourth Hospital of Hebei Medical University, among which 32 CR-ECC were systematically identified and characterized.

### Bacterial identification

Following resuscitation on blood agar plates, all microbial strains were subjected to species identification using the VITEK MS (bioMérieux, France), with *Escherichia coli* ATCC 8739 serving as the standardized calibration strain for instrument validation and quality control throughout the analytical process.

### Antimicrobial susceptibility tests

Antimicrobial susceptibility testing was performed using the Vitek-2 Compact automated microbiology system (bioMérieux, France), with quality control strains *Escherichia coli* ATCC 25,922 and *Pseudomonas aeruginosa* ATCC 27,853 integrated into each analytical run, and results were interpreted according to the CLSI M100-S33 guidelines to ensure standardized, reproducible susceptibility profiling.

### Modified carbapenem inactivation method

A standardized 1 µL inoculum loop was utilized to transfer test colonies from blood agar plates into TSB, followed by 10–15 s of vortex mixing to achieve a homogeneous bacterial suspension. A meropenem disk (10 µg) was fully saturated with the suspension using sterile forceps and incubated at 35 °C for 4 h. Subsequently, the disk was aseptically transferred onto an MH agar plate pre-inoculated with a 0.5 McFarland standard suspension of *Escherichia coli* ATCC 25,922, followed by 18–21 h of incubation at 35 °C for zone of inhibition measurement, adhering to CLSI-recommended mCIM protocols.

### Illumina sequencing

Genomic DNA extracted from the 32 CR-ECC underwent fragmentation using a Covaris ultrasonicator, followed by sequential library preparation steps including end repair, A-tailing, adapter ligation, and purification, with subsequent PCR amplification for library enrichment. Library quality control was rigorously performed using a Qubit 2.0 fluorometer (Thermo Fisher Scientific, America) for concentration quantification and an Agilent 2100 bioanalyzer (Agilent Technologies, America) for fragment size distribution analysis. Qualified libraries were pooled at equimolar concentrations based on validated quantification data and subjected to high-throughput sequencing on the Illumina HiSeq X Ten platform following standardized protocols for whole-genome sequencing of multidrug-resistant pathogens.

### Information analysis

Molecular characterization was performed through an integrated bioinformatics pipeline: MLST was conducted using MLST 2.0 (https://cge.cbs.dtu.dk/services/MLST/) to determine housekeeping gene profiles and STs. Phylogenetic reconstruction was executed in MEGA 11, with resultant trees visualized and annotated via the iTOL web platform (https://itol.embl.de/). Plasmid replicon types were identified using PlasmidFinder 2.1 (https://cge.cbs.dtu.dk/services/PlasmidFinder/) against the *Enterobacteriaceae* database. Antimicrobial resistance genes were analyzed through the Resistance Gene Identifier (RGI) 4.0.3, employing protein homology and mutation models to predict resistance determinants. Virulence gene profiling was achieved by BLAST alignment against the Virulence Factor Database (VFDB), with all analytical workflows standardized to ensure reproducibility and cross-study comparability.

### Ethical approval

All procedures complied with guidelines and were approved by Hebei Medical University Fourth Hospital Ethics Committee (No.2021KS037). The Hebei Medical University Fourth Hospital Ethics Committee also approved the waiver of informed consent to participate in this study owing to its retrospective design. All patient data were anonymised prior to analysis. Study procedures were conducted in accordance with the ethical standards of the Declaration of Helsinki.

## Results

### Distribution of bacterial species

**Fig. 1:**
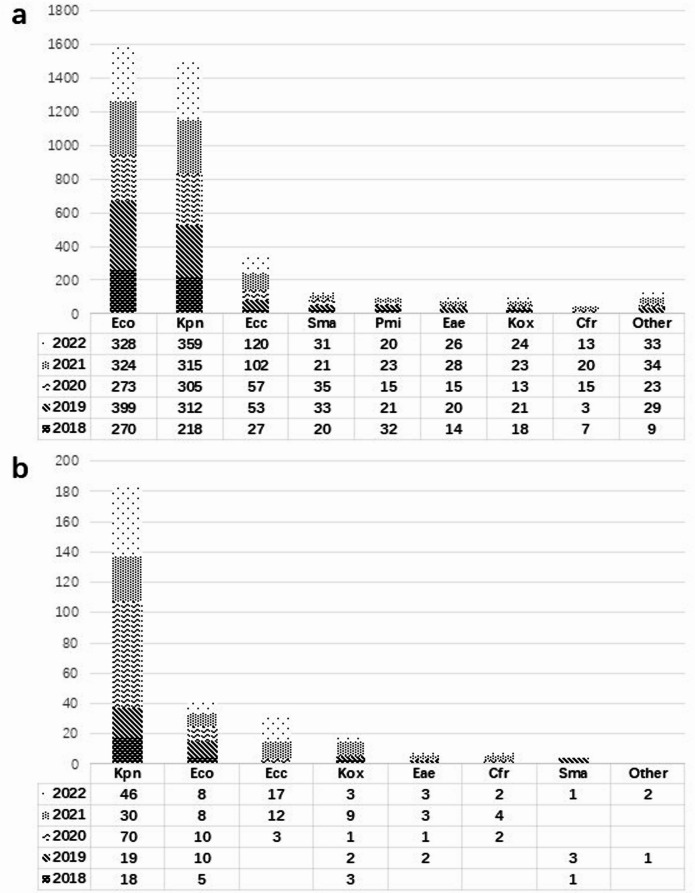
Distribution of *Enterobacteriaceae* from 2018 to 2022. Over the past five years, this study revealed distinct trends in carbapenem-resistant pathogens: The proportion of CRE among total *Enterobacteriaceae* peaked in 2020 at 11.58% (87/751), subsequently declining to 7.42% (66/890) in 2021 and 8.60% (82/954) in 2022. Conversely, CR-ECC demonstrated an inverse trajectory, initially representing 5.26% (3/57) of ECC in 2020 before rising to 11.76% (12/102) in 2021 and 14.17% (17/120) in 2022 (Fig. 2a). Carbapenem-resistant *Klebsiella pneumoniae* maintained dominance throughout the study period, reaching its peak contribution to CRE at 80.46% (70/87) in 2020. Notably, CR-ECC surpassed carbapenem-resistant *Escherichia coli* in 2021, accounting for 18.18% (12/66) of CRE isolates, and further increased to 20.73% (17/82) in 2022, becoming the second most prevalent CRE pathogen after CRKP (56.10%, 46/82) (Fig. 2b).

This five-year surveillance study (2018–2022) identified 4,101 *Enterobacteriaceae*, with *Escherichia coli*(Eco) (*n* = 1,594) and *Klebsiella pneumoniae*(Kpn) (*n* = 1,509) constituting the predominant pathogens, while ECC emerged as the third most prevalent with 359 isolates, demonstrating a consistent annual increase since 2018 (Fig. 1a). Notably, among 299 carbapenem-resistant *Enterobacteriaceae* (CRE) isolates, *Klebsiella pneumoniae* dominated (*n* = 183), followed by the newly recognized carbapenem-resistant ECC (CR-ECC) strains first detected in 2020 and persistently identified through 2022, accumulating 32 cases that positioned CR-ECC as the third most prevalent CRE pathogen after *Klebsiella pneumoniae* and *Escherichia coli* (Fig. 1b).

**Fig. 2:**
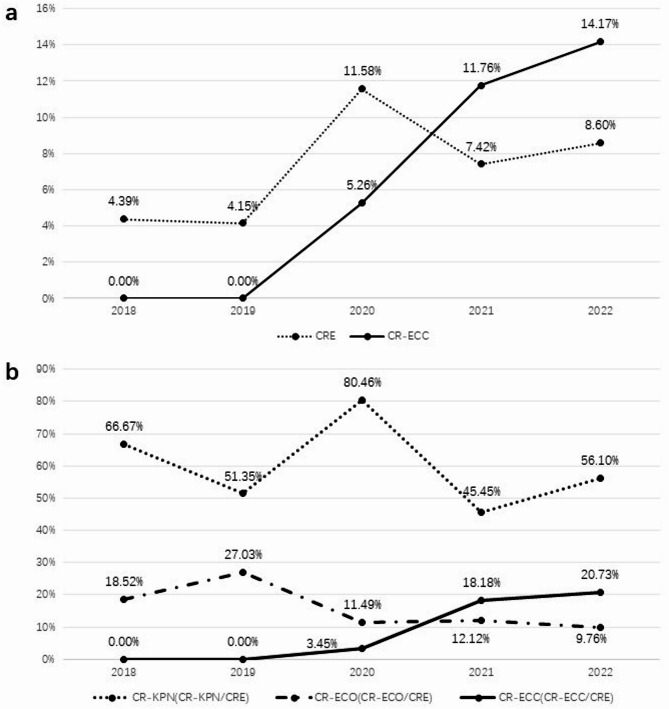
Trends of carbapenem-resistant *Enterobacteriaceae* from 2018 to 2022. The specimen distribution of 32 CR-ECC revealed sputum as the predominant source (43.75%, *n* = 14), followed by blood (25.00%, *n* = 8) and urine (12.50%, *n* = 4), while abdominal fluid and secretion yielded minimal isolates (*n* = 2 each) (Fig. 3a). Departmental distribution analysis demonstrated intensive care units (ICU) as the primary reservoir (53.13%, *n* = 17), with internal medicine (21.88%, *n* = 7) and surgery (18.75%, *n* = 6) constituting secondary hotspots, whereas pediatrics and gynecology accounted for only 3.13% of cases collectively (*n* = 1 each) (Fig. 3b).

**Fig. 3:**
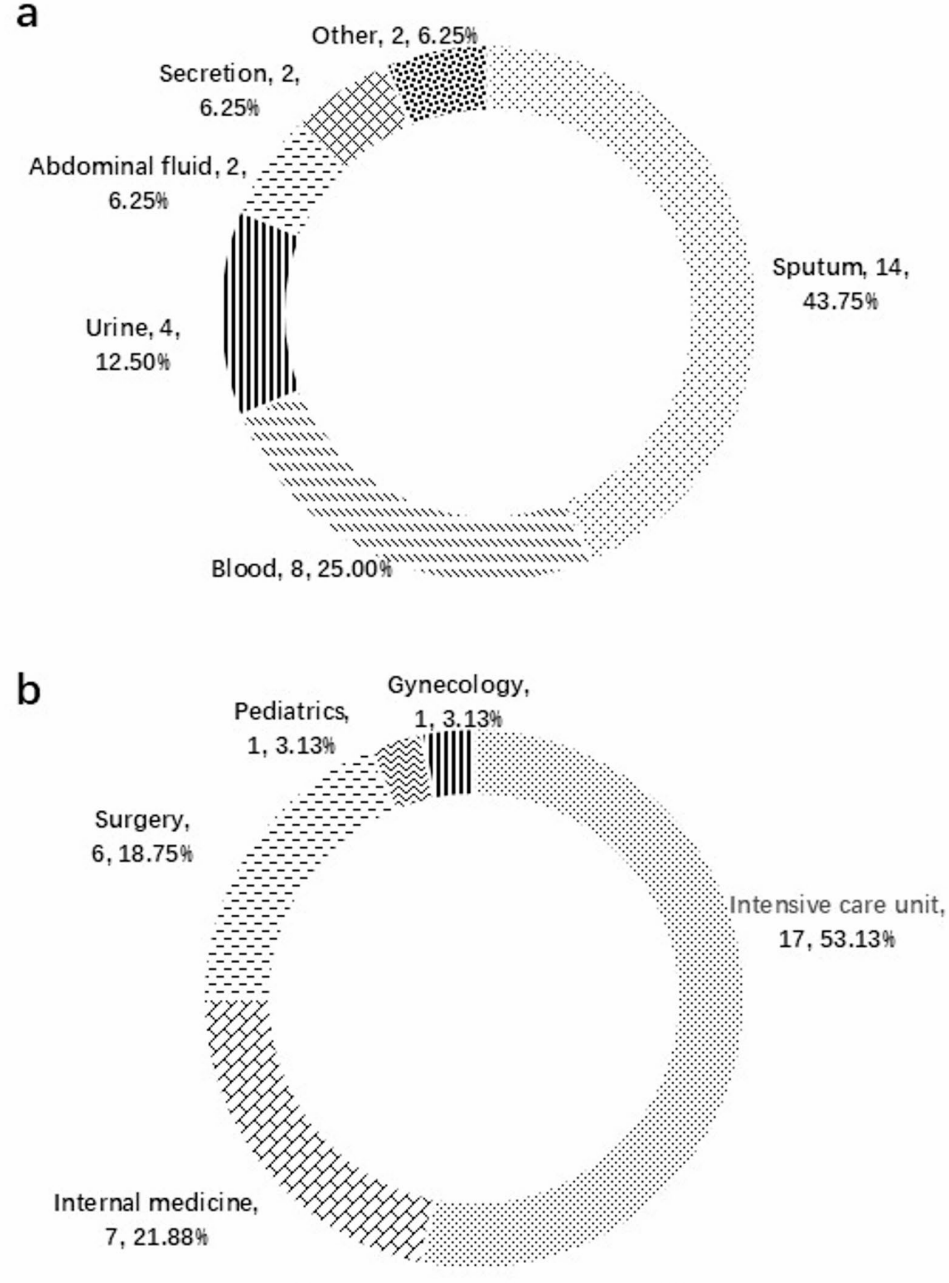
Distribution of carbapenem-resistant *Enterobacter cloacae complexes*.

### Biological information analysis

**Fig. 4:**
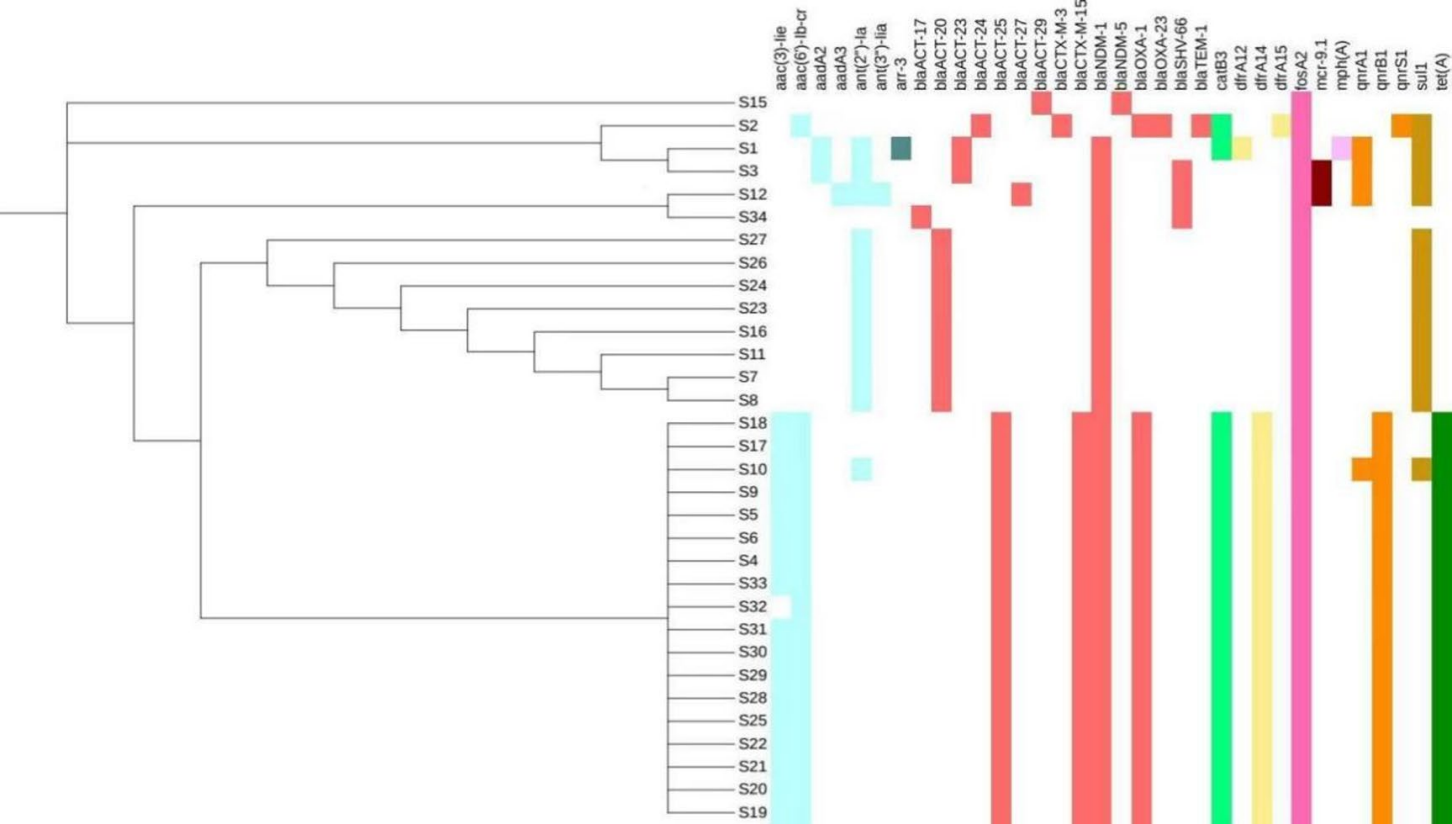
Phylogenetic analysis of carbapenem-resistant *Enterobacter cloacae complexes*.

This comprehensive molecular characterization of 32 CR-ECC revealed that all strains tested positive by the modified carbapenem inactivation method (mCIM), confirming carbapenemase production. Multilocus sequence typing (MLST) based on seven housekeeping genes (*dnaA*,* fusA*,* gyrB*,* leuS*,* pyrG*,* rplB*,* rpoB*) identified eight distinct STs, with ST171 (56.25%, *n* = 18) and ST1077 (25.00%, *n* = 8) predominating, while ST336, ST1740, ST419, ST110, ST35, and ST190 represented minor types (*n* = 1 each). Carbapenemase genotyping showed NDM-1 as the primary resistance determinant, with 18 ST171 co-harboring OXA-1. Notably, one ST1740 lacked NDM but carried OXA-1 and OXA-23, while an ST35 produced NDM-5. The colistin resistance gene mcr-9.1 was detected in two isolates (ST419 and ST110). Plasmid profiling identified five replicon types—Col(pHAD28), IncC, IncHI2/IncHI2A, IncM1, and IncX3—with distribution patterns correlating strongly with ST lineages. All genomic sequences have been deposited in the Sequence Read Archive under BioProject PRJNA1043881, with detailed results summarized in Table 1 and Fig. 4.

**Table 1:** Biological information of 32 CR-ECC isolates

**ID**	**ST type**	**mCIM**	**Carbapenemase/** **Oxacillinase**	**MCR-9.1**	**Plasmid replicons**	**Year**	**Accession**
S1	ST336	+	NDM-1	-	IncHI2/IncHI2A	2020	SAMN38357023
S2	ST1740	+	OXA-1/OXA-23	-	IncM1	2020	SAMN38357024
S3	ST419	+	NDM-1	+	IncHI2/IncHI2A	2020	SAMN38357025
S4	ST171	+	NDM-1/OXA-1	-	Col(pHAD28)	2021	SAMN38357026
S5	ST171	+	NDM-1/OXA-1	-	Col(pHAD28)	2021	SAMN38357027
S6	ST171	+	NDM-1/OXA-1	-	Col(pHAD28)	2021	SAMN38357028
S7	ST1077	+	NDM-1	-	IncC	2021	SAMN38357029
S8	ST1077	+	NDM-1	-	IncC	2021	SAMN38357030
S9	ST171	+	NDM-1/OXA-1	-	Col(pHAD28)	2021	SAMN38357031
S10	ST171	+	NDM-1/OXA-1	-	Col(pHAD28)	2021	SAMN38357032
S11	ST1077	+	NDM-1	-	IncC	2021	SAMN38357033
S12	ST110	+	NDM-1	+	IncHI2/IncHI2A	2021	SAMN38357034
S13	ST35	+	NDM-5	-	IncX3	2021	SAMN38357035
S14	ST1077	+	NDM-1	-	IncC	2021	SAMN38357036
S15	ST171	+	NDM-1/OXA-1	-	Col(pHAD28)	2021	SAMN38357037
S16	ST171	+	NDM-1/OXA-1	-	Col(pHAD28)	2022	SAMN38357038
S17	ST171	+	NDM-1/OXA-1	-	Col(pHAD28)	2022	SAMN38357039
S18	ST171	+	NDM-1/OXA-1	-	Col(pHAD28)	2022	SAMN38357040
S19	ST171	+	NDM-1/OXA-1	-	Col(pHAD28)	2022	SAMN38357041
S20	ST171	+	NDM-1/OXA-1	-	Col(pHAD28)	2022	SAMN38357042
S21	ST1077	+	NDM-1	-	IncC	2022	SAMN38357043
S22	ST1077	+	NDM-1	-	IncC	2022	SAMN38357044
S23	ST171	+	NDM-1/OXA-1	-	Col(pHAD28)	2022	SAMN38357045
S24	ST1077	+	NDM-1	-	IncC	2022	SAMN38357046
S25	ST1077	+	NDM-1	-	IncC	2022	SAMN38357047
S26	ST171	+	NDM-1/OXA-1	-	Col(pHAD28)	2022	SAMN38357048
S27	ST171	+	NDM-1/OXA-1	-	Col(pHAD28)	2022	SAMN38357049
S28	ST171	+	NDM-1/OXA-1	-	Col(pHAD28)	2022	SAMN38357050
S29	ST171	+	NDM-1/OXA-1	-	Col(pHAD28)	2022	SAMN38357051
S30	ST171	+	NDM-1/OXA-1	-	Col(pHAD28)	2022	SAMN38357052
S31	ST171	+	NDM-1/OXA-1	-	Col(pHAD28)	2022	SAMN38357053
S32	ST190	+	NDM-1	-	IncX3	2022	SAMN38357054

### Antimicrobial susceptibility analysis

**Fig. 5:**
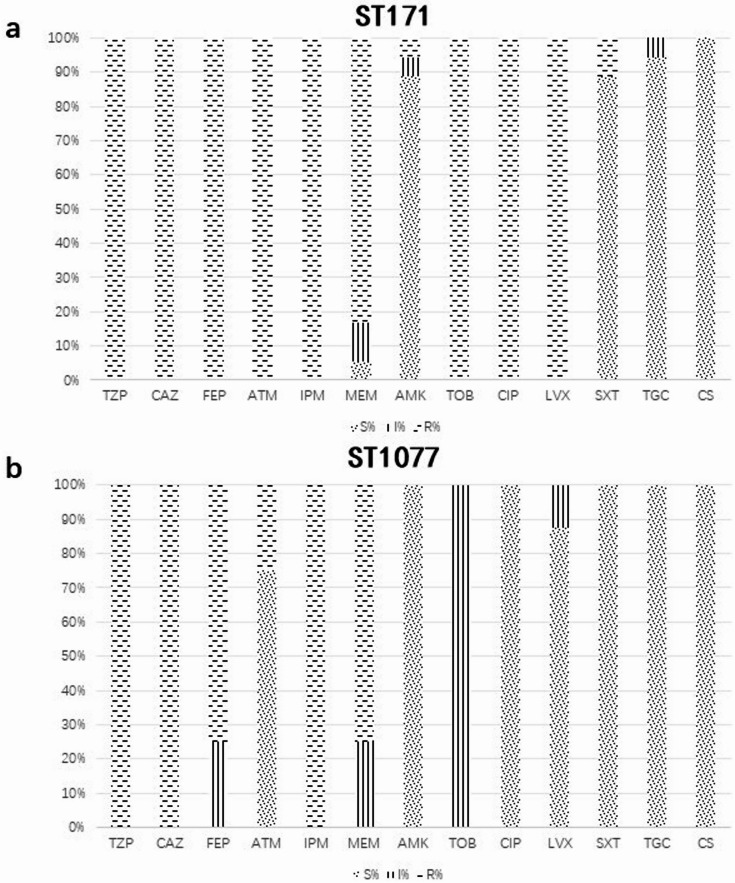
Antimicrobial susceptibility testing results for ST171 and ST1077.

This antimicrobial susceptibility analysis of 32 CR-ECC revealed critical resistance patterns: Universal resistance was observed to β-lactam antibiotics including piperacillin/tazobactam, ceftazidime, and imipenem, with high resistance rates to cefepime (87.50%) and meropenem (84.38%), while aztreonam demonstrated the lowest resistance (78.13%) within this drug class. Among aminoglycosides and fluoroquinolones, amikacin showed exceptional preserved susceptibility, contrasting with tobramycin and fluoroquinolones. Sulfamethoxazole/trimethoprim maintained moderate resistance (12.50%), while no tigecycline/colistin-resistant strains were detected (Table 2 and Fig. 5).

**Table 2:** Antimicrobial susceptibility testing results of 32 carbapenem-resistant Enterobacter cloacae complexes

ID	TZP	CAZ	FEP	ATM	IPM	MEM	AMK	TOB	CIP	LVX	SXT	TGC	CS
S1	64	>=64	8	16	>=16	>=16	<=2	4	<=0.25	1	>=16/304	2	0.5
S2	>=128	>=64	32	>=64	8	>=16	<=2	>=16	>=4	>=8	>=16/304	1	1
S3	64	>=64	32	>=64	>=16	>=16	<=2	4	<=0.25	0.5	<=1/19	2	0.5
S4	64	>=64	16	16	8	8	16	>=16	>=4	>=8	<=1/19	0.5	0.5
S5	64	>=64	16	>=64	4	8	<=2	>=16	>=4	>=8	<=1/19	0.5	0.5
S6	64	>=64	32	16	4	4	8	>=16	>=4	>=8	<=1/19	0.5	0.5
S7	64	>=64	16	<=1	8	8	<=2	8	<=0.25	<=0.25	<=1/19	0.25	1
S8	128	>=64	16	<=1	8	4	<=2	8	<=0.25	<=0.25	<=1/19	0.25	1
S9	>=128	>=64	>=64	>=64	>=16	2	32	>=16	>=4	>=8	<=1/19	0.5	0.5
S10	>=128	>=64	>=64	>=64	>=16	>=16	4	>=16	>=4	>=8	>=16/304	0.5	0.5
S11	128	>=64	16	<=1	8	4	<=2	8	<=0.25	<=0.25	<=1/19	0.5	1
S12	128	>=64	32	>=64	4	4	<=2	4	<=0.25	1	<=1/19	0.5	0.5
S13	>=128	>=64	>=64	<=1	>=16	8	<=2	<=1	<=0.25	<=0.25	<=1/19	2	0.5
S14	>=128	>=64	>=64	>=64	8	4	<=2	8	<=0.25	<=0.25	<=1/19	0.25	1
S15	64	>=64	16	16	4	>=16	8	>=16	>=4	>=8	<=1/19	0.25	1
S16	>=128	>=64	>=64	16	4	4	8	>=16	>=4	>=8	<=1/19	1	0.5
S17	>=128	>=64	>=64	>=64	8	8	4	>=16	>=4	>=8	2/38	2	0.5
S18	64	>=64	>=64	>=64	8	1	8	>=16	>=4	>=8	2/38	4	1
S19	64	>=64	>=64	>=64	8	4	8	>=16	>=4	>=8	<=1/19	0.5	1
S20	>=128	>=64	>=32	>=64	>=16	>=16	4	>=16	>=4	>=8	<=1/19	0.5	0.5
S21	>=128	>=64	4	<=1	8	2	<=2	8	<=0.25	<=0.25	<=1/19	0.25	1
S22	>=128	>=64	4	<=1	8	2	<=2	8	<=0.25	<=0.25	<=1/19	0.25	1
S23	64	>=64	>=64	>=64	>=16	8	4	>=16	>=4	>=8	<=1/19	1	0.5
S24	>=128	>=64	>=64	>=64	4	8	<=2	8	<=0.25	1	2/38	2	2
S25	>=128	>=64	16	<=1	>=16	>=16	<=2	8	<=0.25	0.5	<=1/19	2	0.5
S26	>=128	>=64	>=32	32	>=16	>=16	8	>=16	>=4	>=8	<=1/19	2	0.5
S27	>=128	>=64	>=64	>=64	4	2	8	>=16	>=4	>=8	<=1/19	1	1
S28	>=128	>=64	>=64	>=64	4	8	>=64	>=16	>=4	>=8	4/76	1	0.5
S29	>=128	>=64	>=32	16	>=16	>=16	4	>=16	>=4	>=8	<=1/19	2	0.5
S30	>=128	>=64	>=32	>=64	>=16	>=16	<=2	>=16	>=4	>=8	<=1/19	2	0.5
S31	>=128	>=64	>=32	16	>=16	>=16	8	>=16	>=4	>=8	<=1/19	2	0.5
S32	>=128	>=64	8	>=64	>=16	>=16	<=2	<=1	<=0.25	<=0.12	<=1/19	1	0.5
R%	100%	100%	87.50%	78.13%	100%	84.38%	3.13%	59.38%	59.38%	59.38%	12.50%	0	0

### Analysis of virulence genes

**Fig. 6:**
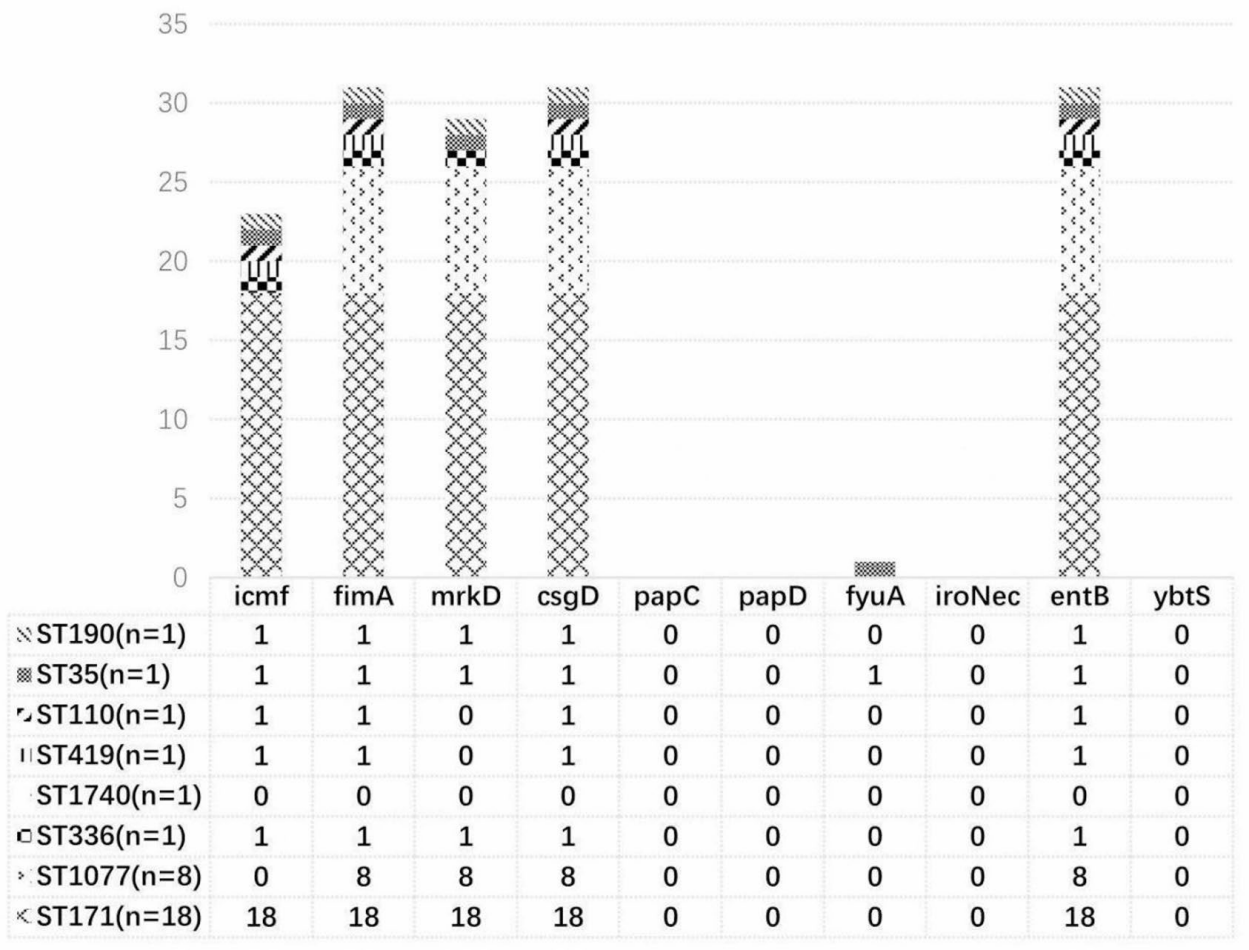
Virulence gene distribution of carbapenem-resistant *Enterobacter cloacae complexes*.

*FimA*, *csgD*, and *entB* were the most prevalent (detected in 31 isolates), followed by *mrkD* (*n* = 29) and *icmf* (*n* = 23), with *fyuA* observed in a single isolate, while no strains harbored *papC*, *papD*, *iroNec*, or *ybtS*. ST-specific profiling revealed conserved virulence patterns in ST171, ST336, and ST190, all carrying *icmf*, *fimA*, *mrkD*, *csgD*, and *entB*. Divergences included the absence of *icmf* in ST1077, loss of *mrkD* in ST419 and ST110, exclusive presence of *fyuA* in ST35, and complete lack of virulence genes in ST1740 (Fig. 6).

## Discussion

According to the 2023 China Antimicrobial Resistance Surveillance System report, *Enterobacter cloacae* remains among the top 10 Gram-negative pathogens, exhibiting resistance rates of 11.3% and 9.0% to imipenem and meropenem, respectively. Our five-year study corroborates its clinical significance, with ECC consistently ranking third among *Enterobacteriaceae* and demonstrating an alarming rise in CR-ECC since their initial detection in 2020. This trend aligns with global observations by Karlsson et al.^1^, who documented increasing carbapenem resistance in *E. cloacae*, underscoring CR-ECC as a critical challenge in anti-infective therapy. While Mauritz et al.^2^ reported ECC’s primary isolation from swabs (42.7%), urine (17.5%), and respiratory secretions (16.1%), our findings highlight respiratory samples (sputum) as the dominant source, particularly within ICUs—a divergence emphasizing CR-ECC’s expanding ecological niche in healthcare settings and its escalating threat to critically ill patients.

Carbapenemase production represents a primary resistance mechanism in CR-ECC, with carbapenemases categorized into serine-based classes A/D (e.g., KPC, OXA) and zinc-dependent metallo-β-lactamases (class B: NDM, VIM, IMP). Among our 32 CR-ECC, 93.75% (*n* = 30) harbored NDM-1, with 56.25% (*n* = 18) co-producing OXA-1. These findings align with Garza et al.^3^, whose multicenter study identified NDM-1 as the predominant carbapenemase gene (80% prevalence) in CRE, reinforcing its global dominance. However, geographical variations emerge in genotype distribution: While Lemonnier et al.^4^ documented rising ECC carbapenem resistance since 2020 linked to VIM surges, our study detected a rare OXA-1/OXA-23 co-occurrence—a notable contrast to Middle Eastern patterns where Malek et al.^5^ reported > 80% prevalence of OXA-48 among CRE. The atypical presence of OXA-23 (traditionally associated with *Acinetobacter baumannii*^6^ alongside OXA-1 in ECC suggests horizontal transfer of resistance plasmids across species boundaries, highlighting an urgent need for surveillance of interspecies resistance gene dissemination.

While Hu et al.^7^ identified ST171 (27.8%) and ST418 (25.0%) as predominant lineages among 36 ECC, and Manandhar et al.^8^ confirmed ST171 dominance in bloodstream infections, global heterogeneity persists—Tian et al.^9^ reported ST51/ST88 as primary types over four years, and Jin et al.^10^ highlighted ST544’s regional prevalence. Our study detected eight STs, dominated by ST171 (*n* = 18), aligning with certain regional data, followed by emerging ST1077 (*n* = 8), a phylogenetically close relative of ST171 that has become a secondary epidemic clone in our setting despite limited global documentation. Minor lineages included singleton ST336, ST1740, ST419, ST110, ST35, and ST190—the latter corroborated by Gartzonika et al.^11^ in their 90 CR-ECC. Plasmid profiling demonstrated ST-replicon associations: ST171 exclusively carried Col(pHAD28), while ST1077 uniformly harbored IncC, patterns suggesting clonal propagation within hospital ecosystems. This contrasts with Halder et al.^12^, who identified IncFIB, IncFII, IncX3, IncHI1-HI2, IncC, and IncR as predominant replicons across eight hospitals. Notably, Han et al.^13^ reported IncHI2/IncHI2A dominance (64.7%), whereas our three IncHI2/IncHI2A isolates (ST336, ST419, ST110) included two *mcr-9.1* carriers (ST419, ST110), supporting Xu et al.’s^14^ findings on IncHI2/IncHI2A’s role in stabilizing *mcr-9* and *NDM-1* co-occurrence. Additional replicon patterns included IncX3 in ST35/ST190—a key vector for carbapenemase dissemination in China^15^—and IncM1 in ST1740, a rare CR-ECC-associated replicon linked to serine carbapenemase acquisition^16^.

CR-ECC harbor diverse resistance determinants beyond carbapenemase genes, including AmpC β-lactamases (ACT-20, ACT-25), extended-spectrum β-lactamases (CTX-M-15, SHV-66), aminoglycoside-modifying enzymes (AAC(3)-Iie, ANT(2’’)-Ia), fluoroquinolone resistance mechanisms (AAC(6’)-Ib-cr, QnrB1), fosfomycin-inactivating FosA2, and colistin resistance gene mcr-9.1. Our findings demonstrate ST-specific resistance gene profiles, enabling tailored antimicrobial strategies through clone-targeted susceptibility tracking. Notably, tigecycline and colistin retained robust activity (100% susceptibility) despite mcr-9.1 detection, with no observed MIC elevation—a phenomenon corroborated by Lumbreras et al.^17^, though Wu et al.^18^ warned of colistin-induced mcr-9 upregulation and MIC drift, necessitating vigilant susceptibility monitoring. Rujirat et al. reported that mcr-8- and mcr-9-positive CRKpnC exhibited colistin resistance mediated jointly by plasmid-borne mcr and chromosomal mutations^19^. Amikacin also exhibited high efficacy, aligning with Kurihara et al.’s^20^ report on its sustained potency against carbapenem-nonsusceptible *Enterobacteriaceae*. Although *The Sanford Guide to Antimicrobial Therapy* (50th edition) recommends aztreonam/ceftazidime-avibactam for MBL producers, our NDM-dominant CR-ECC showed 78.13% aztreonam resistance. This resistance was mediated by co-produced ESBLs/AmpC β-lactamases, as supported by the ST171 clone with blaNDM-1 and blaCTX-M-15. Li et al.^21^ reported 100% synergy with aztreonam/ceftazidime-avibactam against MBL-producing ECC, supporting combination therapy. Thus, aztreonam susceptibility should be interpreted with coexisting ESBL/AmpC genes.

Virulence genes encoding adhesins, iron acquisition systems, and other pathogenic determinants, typically located on pathogenicity islands, plasmids, and mobile genetic elements, play dual roles in enhancing bacterial survival and modulating antimicrobial resistance expression, making their characterization critical for understanding pathogenicity-resistance interplay. This study identified six virulence genes, predominantly *fimA*, *csgD*, and *entB*, followed by *mrkD* and *icmf*. Yan et al.^22^ demonstrated *csgD*’s dual function in promoting biofilm formation and regulating *fimA* expression, while Han et al.^23^ linked *entB* to enhanced siderophore production and serum resistance. Unlike *Klebsiella pneumoniae* lineages described by Hu et al.^24^(*fimH/mrkD/entB/wzi* clusters) or *NDM*-positive ECC with elevated *clpB/icmf/VasD-Lip/acrA* reported by Yu et al.^25^, our isolates exhibited distinct profiles, underscoring interspecies and geographical variability in virulence gene distribution. Notably, *fyuA*, detected exclusively in ST35, aligns with Lin et al.’s^26^ discovery of a *Yersinia* high-pathogenicity island harboring *fyuA* on the chromosome of an MCR-9/NDM-1 co-producing *E. cloacae*, suggesting rare horizontal acquisition events in specific genetic backgrounds.

The rising prevalence of CR-ECC over the past five years underscores the critical need for genomic surveillance. This study elucidates regional distribution patterns of carbapenemase genes and co-occurring resistance determinants, tracks dynamic shifts in dominant STs, and maps antimicrobial susceptibility trends, while simultaneously investigating virulence gene heterogeneity to expand China’s molecular epidemiological database for CRE. By correlating ST-specific resistance profiles with clinical susceptibility data, we aim to refine antimicrobial stewardship protocols, strengthen hospital infection control frameworks, and inform targeted interventions, ultimately disrupting transmission networks of high-risk clones like ST171 and emerging lineages such as ST1077. These efforts collectively address the dual challenge of rising resistance and pathogenic adaptability, providing actionable insights to mitigate the public health threat posed by CR-ECC dissemination.

## Data Availability

The sequencing data presented in the study are deposited in the National Library of Medicine repository, accession number PRJNA1043881.( https://www.ncbi.nlm.nih.gov/bioproject/PRJNA1043881/).
